# YOLOX target detection model can identify and classify several types of tea buds with similar characteristics

**DOI:** 10.1038/s41598-024-53498-y

**Published:** 2024-02-03

**Authors:** Mengdao Yang, Weihao Yuan, Gaojian Xu

**Affiliations:** 1 Anhui Agricultural University, Hefei, 230036 Anhui China; 2 School of Information and Artificial Intelligence, Anhui Agricultural University, Hefei, 230036 China

**Keywords:** Plant sciences, Computer science, Image processing

## Abstract

Currently, the accuracy of tea bud identification is crucial in the intelligent development of the tea industry, and this is due to the fact that identifying tea buds is a key step in determining the quality of tea and distinguishing the categories. In this experiment, 3728 tea shoots with similar characteristics in four categories (Anji White Tea, Huangshan Seed, Longjing 43, and NongKang Early) were photographed to establish the dataset TBD (Tea Bud Dataset). In this experiment, we constructed a tea shoot recognition model. We used seven mainstream algorithms (YOLOv4, YOLOv5, YOLOX, YOLOv7, EfficientDet, Faster R-CNN and CenterNet) to conduct shoot recognition comparison experiments and found that the YOLOX algorithm performs the best with its Precision, Recall, F1 score, mAP 89.34%, 93.56%, 0.91, and 95.47%, respectively. Then the YOLOX algorithm combined with the dataset to construct the shoot recognition model, the shoots of four kinds of tea to establish a tea shoot classification model, the model to identify the Anji white tea shoots of Precision 76.19%, the yellow mountain species of Precision 90.54%, Longjing 43 Precision 80%, NongKang early to the morning of the Precision was 77.78%. The results of this experiment show that the established tea shoot classification model has achieved a better classification of the above four types of tea shoots, which can also understand the feasibility of mechanical intelligent tea picking and provide some theoretical support for the application of mechanical intelligent tea picking in practice.

## Introduction

Tea bud plucking is one of the critical factors limiting the development of the tea industry. In the process of tea production, tea bud plucking is a key step in determining the quality of dry tea^1^. Tea-drinking groups of tea quality requirements are getting higher and higher. China’s tea industry needs to adapt to the changes in market demand, the source of which will be to grasp the tea bud plucking this step. The current tea bud plucking is the main way of artificial tea plucking and mechanical tea plucking. Artificial plucking of tea buds of high quality to meet different standards of picking, bud integrity rate is high. However, because of its high cost, low speed and easy to miss the tea picking period, the research related to mechanical tea picking is on the agenda. The limitation of the current mechanical tea picking is the low rate of bud integrity. With the help of computer vision technology, it can identify the tea shoots, types and stalks to improve the mechanical tea-picking shear further shoots^2,3^.

Tender bud-picking faces, in addition to the mechanization problem, there is also the problem of intelligence. The realization of intelligent tea-picking machines can be more adaptable to the current tea production environment. In recent years, the application of computer vision technology in agriculture, pest detection, crop growth monitoring, and other aspects of computer vision technology has also been used by researchers in tea shoot picking and has achieved many excellent results. Computer vision technology in shoot recognition research can be divided into two stages: the traditional image recognition stage and the deep learning recognition stage. The traditional image recognition stage is mainly based on traditional image processing algorithms, image segmentation and edge detection algorithms to recognize tea shoots. Jiang et al.^4^ determined the threshold value of image segmentation by analyzing the histogram of the greyscale image. Finally, they constructed an image detection algorithm for tea shoots based on color factor and image fusion algorithm. Shao^5^ used the texture, color and shape of the shoots as the recognition features of tea shoots, and the final trained model can classify tea shoots into four types: one shoot, one shoot and one leaf, one shoot and two leaves and one shoot and three leaves. Wu et al.^6^ improved the maximum variance automatic thresholding method based on the pixel information of the G-B and G components of the shoot image and then recognized the tea shoots by the improved maximum variance automatic thresholding method. Long et al.^7^ extracted the super green features of shoot images by super green features an OSTU image segmentation algorithm and accurately located the picking points in shoot images by combining the edge detection and skeletonisation processed shoot picking point localization algorithm. Wang et al.^8^ used the OTSU image segmentation algorithm, G-B color features and morphological processing to increase the color difference between old leaves and shoots and combined with the SSD target detection algorithm to train the tea shoot recognition model, and finally achieved the target detection of tea shoots. Zhang et al.^9^ used the Yolov3^10^ target detection algorithms with an optimized SSP module to construct a tea shoot recognition model. Xu et al.^11^ used three convolutional neural networks, ResNet50^12^, ResNet101^13^, and VGG^14^, respectively, as the backbone feature extraction network of the Faster R-CNN^15^ target detection algorithms to build a tea shoot recognition model and by comparing the SSD^16^ algorithm to build a tea shoot recognition model, it was concluded that It was concluded that the tea shoot recognition model established by Faster R-CNN algorithm is better than the tea shoot recognition model established by SSD algorithm. The accuracy of the tea shoot recognition model established by Joao Neto et al.^17^ and others based on the GooLeNet^18^ algorithm is higher. Compared to recognizing tea shoots through traditional image processing algorithms such as image segmentation and edge detection, the accuracy of the tea shoot recognition model established by the target detection algorithm is higher than that of the traditional algorithm recognition and has better robustness.

To achieve intelligent harvesting of tea shoots, it is key to achieve accurate identification and classification of tea shoots. The emergence of computer vision technology makes it possible to identify tea shoots accurately. After years of research and application, the following problems and difficulties mainly exist in the identification and classification of tea shoots:

1. The accuracy of the target detection algorithm based on deep learning to identify tea leaf shoots is generally not high.
2. There are fewer studies on tea shoot classification, but tea shoot classification is necessary for practical production. The study of the tea shoot classification model is of great significance to improve the ability of tea quality identification, promote scientific research and innovation, and promote the intelligent development of the tea industry.

Combining the above problems and difficulties, the main research content of this thesis is as follows:

1. Research on tea shoot image recognition, using YOLOv4^19^, YOLOv5^20^, YOLOX^21^, YOLOv7^21^, EfficientDet^22^, Faster R-CNN and CenterNet^23^ seven target detection algorithms to establish the tea shoot recognition model for comparative experiments, and comprehensively assess the tea shoot recognition model established by each algorithm. Shoot recognition model established by each algorithm.
2. Study the image classification of tea leaf shoots based on target detection algorithms. The best-performing algorithm in (1) is used in combination with the dataset TBD to establish the tea leaf shoot classification model.

## Methods

### Dataset construction

**Figure 1:**
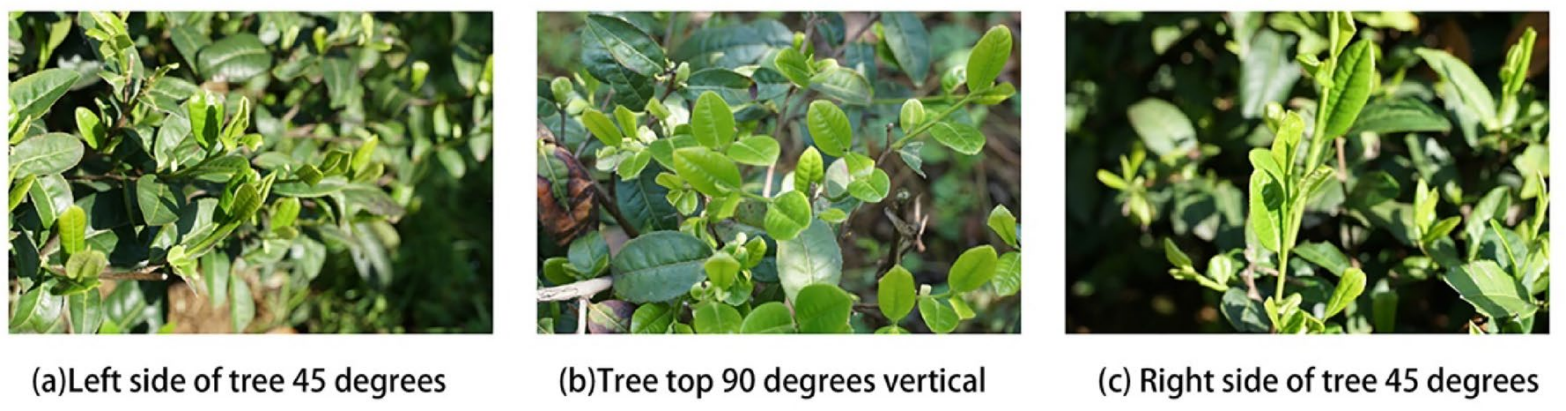
Shoots taken from different angles.

Tea shoot recognition based on a convolutional neural network requires a large number of tea shoot pictures as sample images to be input into the convolutional neural network to train the network parameters, and theoretically, the more tea shoot pictures are trained, the more robust the model^24^. In this experiment, we travelled to the high-tech agricultural park in Dayang Town, Hefei City, Anhui Province, to take pictures of the shoots, and the whole shooting process had both cloudy and sunny days, with both good and bad lighting conditions. In view of the current shape of the tea tree, it is mainly pruned as a dome shape in order to ensure that the distance from the camera to the tea shoots is the same as well as to shoot the tea shoot images from different angles, this experiment chose to shoot the tea shoot images from three angles, taking the cross-section of the tea tree as the reference, the three angles are 90° vertical at the top of the tea tree, 45° tilted on the left side of the tea tree, and 45° tilted on the right side of the tea tree. The images of tea shoots captured from these three angles are shown in Fig. 1.

A total of 3728 images of tea shoots were taken during the whole filming process, including four tea varieties: “Longjing 43”, “NongKang Early”, “Anji White Tea”, and “Huangshan Seed”.

### YOLOv4

Alexey Bochkovskiy, Chien-Yao Wang, and Hong-Yuan Mark Liao proposed the YOLOv4 algorithm based on further summarizing the previous research results. The authors of YOLOv4 divide the target detector into four parts: input, backbone, neck, and head. Input is used to receive the image data; backbone is used to pre-train the image input; neck is used to collect different feature maps; and head is used to predict the feature maps. It is used to receive the image data; the backbone pre-trains the image coming in from input; the neck is used to collect different feature maps; and the head is used to predict the feature maps. When optimizing the algorithm, the authors of YOLOv4 also took into account the input network resolution, the number of convolutional layers, the number of parameters, and other factors. Finally, they determined the overall architecture of YOLOv4 after many adjustments and experiments.

### YOLOv5

YOLOv5 is further improved on the basis of YOLOv4. YOLOv5 adds the Mosaic data enhancement method, adaptive anchor frame calculation and adaptive image scaling in the input part, and the Mosaic data enhancement method can enrich the dataset, improve the training speed of the network and reduce the memory requirement of the model. In order to deal with the large amount of redundant information, YOLOv5 also proposed an adaptive image scaling method that can adaptively scale the image to solve this problem. In the backbone section, some new ideas from other target detection algorithms are incorporated; in the Neck section, the fusion ability of the network features is enhanced; in the head section, YOLOv5 uses the improved GIOU_Loss function, which just solves the problem of optimizing for the situation where the prediction frame and the target frame do not intersect, and the inability to distinguish between two prediction frames of the same size and the same target frame, in the case where the two prediction frames have the same size and the same IOU cannot be distinguished.

YOLOv5 has four versions: YOLOv5_s, YOLOv5_m, YOLOv5_l and YOLOv5_x, and YOLOv5_s is used in this experiment.

### YOLOX

YOLOX is proposed by Zheng Ge et al. It fully learns and borrows from the hot anchorless detection, advanced label assignment strategy and end-to-end detector in the field of target detection in recent years and adds four components of Decoupled Head, Data Augmentation, Free Anchor, and SimOTA sample matching to Yolov3 to build an end-to-end target detection framework without anchors. The concept of anchors in the field of target detection has helped target detection algorithms to improve their accuracy, but at the same time, anchors also make detection more complex. Anchorless has developed rapidly in the field of target detection in recent years. Numerous studies have shown that the performance of anchorless detectors can be comparable to that of anchored detectors. The anchorless mechanism makes the training and decoding phases of the detectors simpler. The anchorless mechanism added to YOLOX significantly reduces the parameters of YOLOX and also makes the recognition accuracy and detection speed of YOLOX faster.

### YOLOv7

YOLOv7 is a deep-learning neural network for real-time target detection, proposed in early 2023 by Alexey Bochkovskiy, Chien-Yao Wang, and Hong-Yuan Mark Liao^25^ at American University.YOLOv7 is the latest version of the YOLO family of algorithms, which, compared to previous versions, has improved both in terms of accuracy and speed.FPN is the enhanced feature extraction network of YOLOv7. The three adequate feature layers obtained in the backbone part are subjected to feature fusion in this part. The purpose of feature fusion is to combine feature information at different scales. In the FPN part, the already obtained adequate feature layers are used to continue the feature extraction. The structure of PANet is still used in YOLOv7. PANet aggregates features at different levels through a top-down and bottom-up bi-directional fusion network, which not only up-samples the features to achieve feature fusion but also down-samples the features again to achieve feature fusion. Yolo Head is the classifier and regressor of YOLOv7. Backbone and FPN, we have obtained three enhanced adequate feature layers. Each feature layer has a width, height, and number of channels. At this point, we can view the feature map as a collection of feature point after feature point, with three a priori frames on each feature point and each a priori frame with several channels for each feature. YOLO Head makes a judgment on the feature points and determines whether the a priori frames on the feature points have an object that corresponds to them. As with previous versions of YOLO, the decoupled heads used in YOLOv7 are together, i.e., classification and regression are implemented in a single 11-convolution. Thus, the work done by the entire YOLOv7 network is feature extraction-feature enhancement-prediction of the object situation corresponding to the a priori box^26^.

### Faster R-CNN

Faster R-CNN was proposed by Ren et al.^25^, which is further optimised and improved based on R-CNN and Fast R-CNN. Faster R-CNN consists of two parts: a region proposal network (RPN) and a Fast R-CNN combined with an RPN. RPN is one of the most extensive improvements of Faster R-CNN, which draws on the neural network mechanism of “attention” proposed by Chorowski et al.,^27^ where the RPN informs the Fast R-CNN in advance of the Fast R-CNN and the Fast R-CNN. RPN is a fully convolutional neural network that takes as input the feature map output from the CNN network. Each pixel in the feature map can be used as an anchor point in the RPN. Each anchor point generates 9 default-sized candidate frames, and a convolution operation is performed on each of them to generate k candidate regions, each of which is assigned to a candidate region, and each of which is assigned to a candidate region. Each candidate region is assigned a corresponding class label with positive samples (1) and negative samples (0).

### EfficientDet

EfficientDet was proposed by Mingxing Tan, Quoc V.Le of the Google team in 2019, which simultaneously scales the depth, width and image resolution of the network according to certain rules to achieve network performance improvement. In order to determine the scaling relationship between network depth, width and image resolution, EfficientDet authors made an abstraction of the whole convolutional neural network. Based on the mathematical model of the convolutional neural network, the authors of EfficientDet started repeated experiments. They came to the following conclusion: scaling up any of the dimensions of width, depth, and resolution can improve the performance of the network, but after scaling up to a specific magnification, the performance improvement is not apparent, and after that, it is necessary to balance the magnification of the width, depth and resolution scaling. Based on the above findings, the authors propose compound scaling, which balances the scaling multiplicity of width, depth and resolution by mixing the coefficients.

There are eight versions of EfficientDet, from EfficientDet-B0 to EfficientDet-B7, and EfficientDet-B0 is used in this experiment.

### CenterNet

CenterNet is an anchor-free target detection algorithm proposed by Xingyi Zhou, Dequan Wang, Philipp Krahenbuhl, et al. in 2019.CenterNet is an improvement on CornerNet, which is different from previous regression-based target detection algorithms and candidate region-based target detection algorithms. CenterNet does not have the concept of an anchor point, instead of a centre point.CenterNet network structure mainly consists of a feature extraction network and prediction module. The prediction module consists of three branches of a convolutional neural network, which are used to predict the heatmap, the target's centroid coordinate and the target's width and height, respectively (Table 1).

**Table 1:** Training parameters when building tea bud identification models using different algorithms.

Training parameter settings								
Parameter name	Type	YOLOv4	YOLOv5	YOLOX	YOLOv7	Faster R-CNN	EfficientDet	CenterNet
Anchor_mask	List	[[6,7,8],[3,4,5],[0,1,2]]	–	–	–	–	–	–
Anchor_size	List	–	[[8,16,32],[3,4,5],[6,1,2]]	–	–	[8,16,32]	–	–
Input_shape	List	[416,416]	[640,640]	[640,640]	[640,640]	[512,512]	[512,512]	[512,512]
Backbone	String	–	–	–	ELAN	ResNet50	–	ResNet50
Freeze_epoch	Int	150	150	150	150	150	150	150
Freeze_batch_size	Int	8	16	8	8	16	16	16
Freeze_lr	Float	0.001	0.001	0.001	0.001	0.001	0.001	0.001
UnFreeze_epoch	Int	300	300	300	300	300	300	300
UnFreeze_batch_size	Int	4	8	4	4	8	8	8
Unfreeze_lr	Float	0.0001	0.0001	0.0001	0.0001	0.0001	0.0001	0.0001

The anchor-mask is utilized to determine the a priori frame of the feature map. The input-shape denotes the dimensions of the input image. The process of model training consists of two distinct phases, namely the freezing phase and the unfreezing phase. In the freezing phase, a total of 150 epochs are executed. The batch_size and learning rate values can be found in the aforementioned table. Both the backbone and feature extraction networks' parameters remain fixed during the freezing phase. Only the networks other than the backbone and feature extraction networks undergo fine-tuning in the unfreezing phase. The batch_size and learning rate values can be found in the aforementioned table. Additionally, the parameters of the feature extraction networks remain fixed during the freezing phase. During the freezing training period, the parameters of both the backbone network and the feature extraction network remain fixed. Only the remaining parts of the network are fine-tuned. The unfreezing phase consists of a total of 150 epochs, with the batch size and learning rate specified in the aforementioned table. In this phase, both the backbone network and the feature extraction network are unfrozen, resulting in changes to all parameters of the network. The training process consists of two phases, spanning a total of 300 epochs. During each training epoch, the network weights are saved as a file. Ultimately, the last epoch is selected, along with the loss value associated with the smallest network weights file. It is important to note that the quality of the network model cannot be solely judged based on the loss value. Instead, the magnitude of the loss value is used to determine whether the network model has converged during training. Specifically, when the loss value exhibits minimal fluctuations within a certain range, it indicates that the network model has converged and the training process can be terminated.

### Evaluation methodology

In this experiment, a total of six indicators, namely Precision, Recall, F1 score, mAP, training time and FPS, were used to evaluate the models. Because it is often difficult to compare who is good and who is terrible when using multiple indicators to do an evaluation, in the establishment of the tea shoot recognition model experiment using the indicator weight method to analyze the good and bad of each model comprehensively, the formula for the comprehensive indicators is as follows.

$$\begin{array}{c}\left\{\begin{array}{c}y={w}_{p}\times Precision+{w}_{r}\times Recall+{w}_{f}\times F1\times 100\%+{w}_{m}\times mAP\\ {w}_{p}+{w}_{r}+{w}_{f}+{w}_{m}=1\end{array}\right.\end{array}$$

where ${\text{w}}_{\text{p}}\text{,} \, {\text{w}}_{\text{r}}\text{,} \, {\text{w}}_{\text{f}}\text{,} \, {\text{w}}_{\text{m}}$ corresponds to the weights of Precision, Recall, F1 score and mAP, respectively. Given that both Precision and Recall have the problem of a single value being immense, which cannot reflect the model accuracy comprehensively, the weighting should be lowered in the evaluation of comprehensive indexes. The F1 score is the reconciled average of Precision and Recall, which is more reflective of the overall accuracy of the model, and the weighting should be slightly higher in the evaluation of comprehensive indexes. mAP is the Precision area under the curve, which is the primary evaluation index of the target detection algorithm. It can accurately reflect the overall accuracy of the model, is the primary evaluation index in the target detection algorithm, and should have the highest weight in the evaluation of comprehensive indexes.

## Results

A comparison of the accuracy metrics of the models trained using the seven algorithms YOLOv4, YOLOv5, YOLOX, YOLOv7, EfficientDet, Faster R-CNN and CenterNet in combination with the TBD dataset is shown in Table 2.

**Table 2:** Comparison of precision indexes for each algorithm training.

Evaluation metrics	YOLOv4	YOLOv5	YOLOX	YOLOv7	EfficientDet	Faster R-CNN	CenterNet
Precision	93.39%	90.09%	89.34%	89.39%	88.84%	62.79%	92.91%
Recall	90.99%	89.70%	93.56%	93.99%	92.27%	97.21%	87.12%
F1 score	0.92	0.89	0.91	0.92	0.91	0.76	0.90
mAP	94.30%	94.21%	95.47%	94.59%	93.74%	93.45%	93.53%

The combined metrics of the seven algorithms under the TBD dataset are shown in Table 3.

**Table 3:** Comprehensive index comparison of 6 algorithms under TBD dataset.

Evaluation metrics	YOLOv4 (%)	YOLOv5 (%)	YOLOX (%)	YOLOv7 (%)	EfficientDet (%)	Faster R-CNN (%)	CenterNet (%)
y0.25	92.67	90.75	92.34	92.49	91.46	82.36	90.89
y0.5	93.18	91.78	93.25	93.23	92.28	85.52	91.76
y0.6	93.41	92.30	93.77	93.49	92.55	87.27	92.12
y0.7	93.64	92.82	94.21	93.75	92.82	89.01	92.47

In Table 3, the values of ${{\text{w}}}_{{\text{p}}}$, ${{\text{w}}}_{{\text{r}}}$, ${{\text{w}}}_{{\text{f}}}$, and ${{\text{w}}}_{{\text{m}}}$ are 0.25 respectively for y0.25. For y0.5, ${{\text{w}}}_{{\text{p}}}$, ${{\text{w}}}_{{\text{r}}}$, ${{\text{w}}}_{{\text{f}}}$, and ${{\text{w}}}_{{\text{m}}}$ are 0.1, 0.1, 0.3, and 0.5 respectively. Similarly, y0.6 indicates that ${{\text{w}}}_{{\text{p}}}$, ${{\text{w}}}_{{\text{r}}}$, ${{\text{w}}}_{{\text{f}}}$, and ${{\text{w}}}_{{\text{m}}}$ are 0.1, 0.1, 0.2, and 0.6 respectively, and y0.7 indicates that ${{\text{w}}}_{{\text{p}}}$, ${{\text{w}}}_{{\text{r}}}$, ${{\text{w}}}_{{\text{f}}}$, and ${{\text{w}}}_{{\text{m}}}$ are 0.1, 0.1, 0.1, and 0.7 respectively.

**Figure 2:**
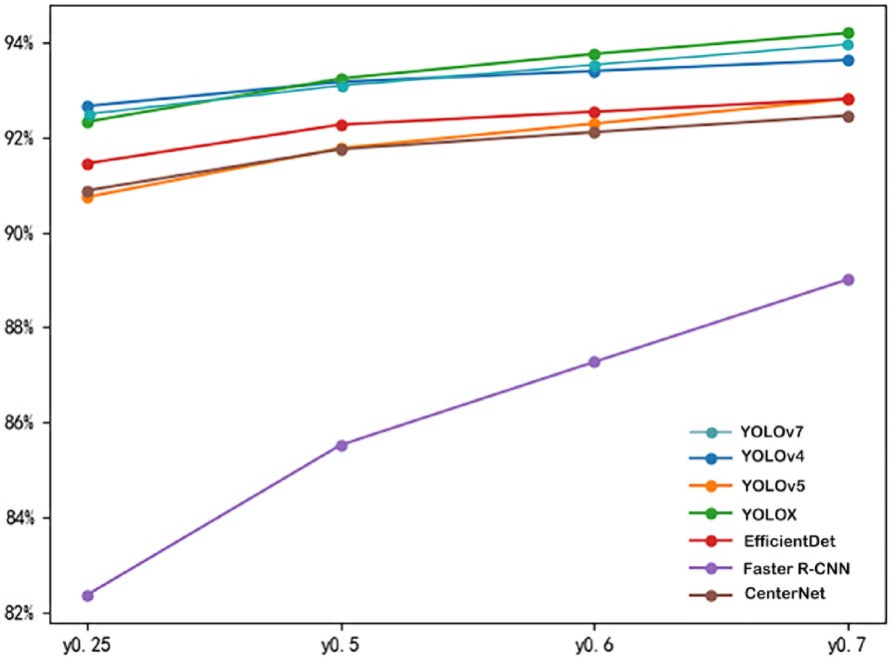
Comprehensive index comparison of 7 algorithms.

The comprehensive index comparison of the seven algorithms is shown in Fig. 2.

The YOLOX algorithm was tested against six groups of mainstream algorithms, which proved that the YOLOX algorithm had the best performance among them. Therefore, the classification model will continue to be built by the YOLOX algorithm.

**Figure 3:**
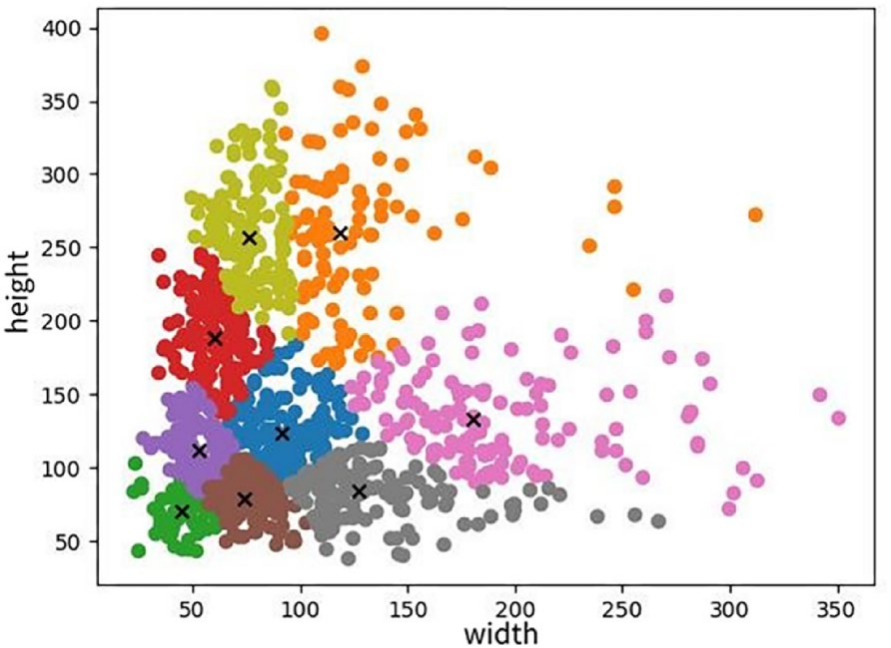
K-means clustering boxes in TBD dataset.

The parameter configurations of YOLOX can be found in Table 1. Compared with the parameter configurations of YOLOX in the shoot recognition model, the batch size in this experiment is increased to make full use of the GPU's arithmetic power and accelerate the model building. This experiment uses K-means to optimize the position of the prior frames before the model starts training. The widths and heights of the nine a priori boxes obtained by K-means are shown in Fig. 3.

**Figure 4:**
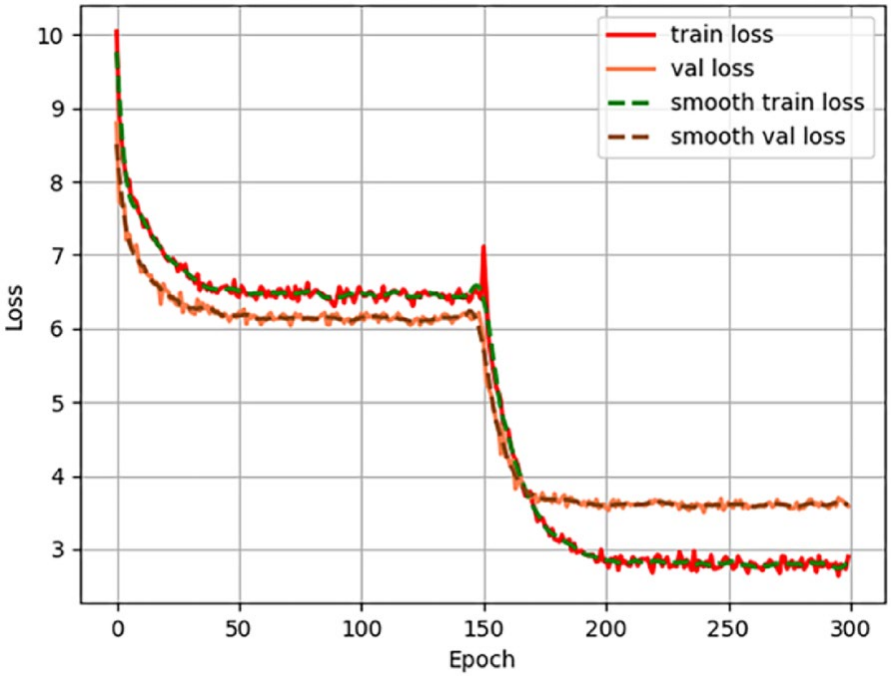
Change of loss value of YOLOX classification model.

The variation of loss values of YOLOX modelled tea shoot classification is shown in Fig. 4.

**Figure 5:**
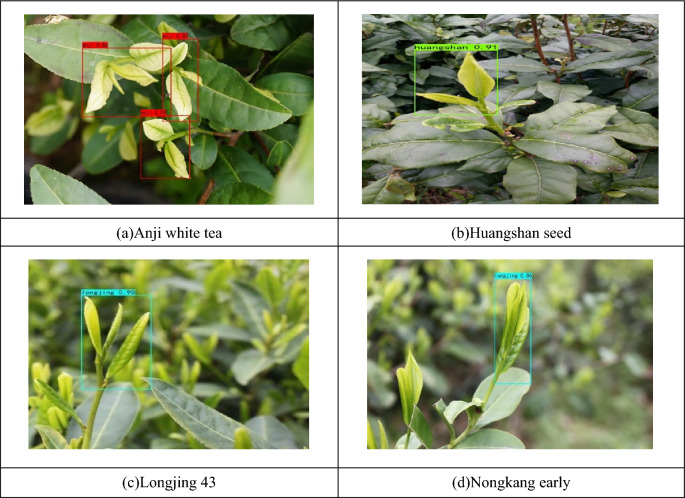
YOLOX classification model identifies tea sprouts of different varieties.

As can be seen in Fig. 4, YOLOX has a convergence process in both training phases and both converge at about 50 iterations. The results of YOLOX classification model for recognizing tea shoots are shown in Fig. 5.

From Fig. 5, it can be seen that the YOLOX classification model is able to identify the shoots and their varieties. For multi-target images with multiple shoots, YOLOX can also identify and classify multiple shoot images. However, YOLOX identifies NongKang Early as Longjing shoots in Fig. 5d, which indicates that there are misclassifications in the YOLOX classification model as well.

**Figure 6:**
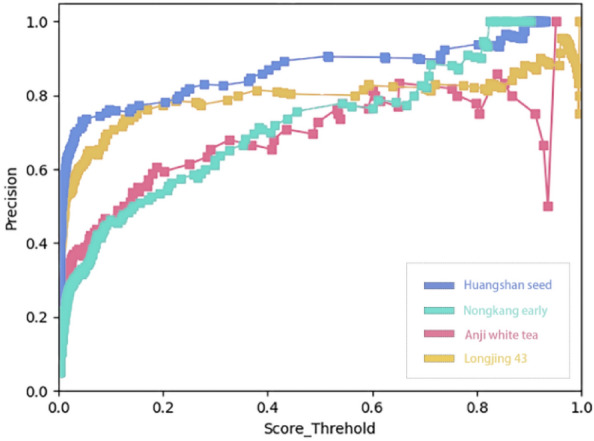
Precision metrics for YOLOX classification models.

The Precision of YOLOX classification model is shown in Fig. 6.

**Figure 7:**
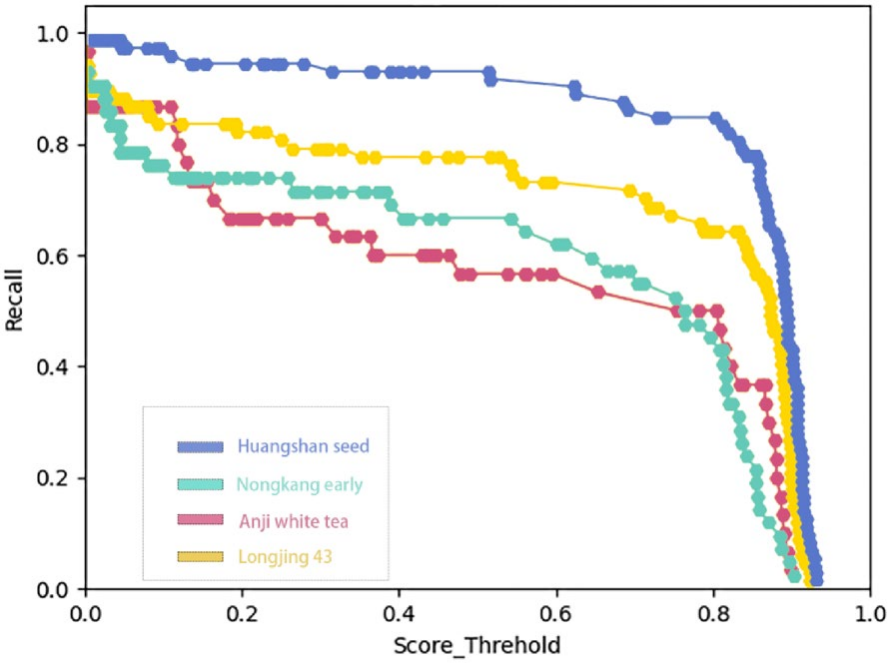
Recall metrics for YOLOX classification models.

Recall of the YOLOX classification model is shown in Fig. 7.

**Figure 8:**
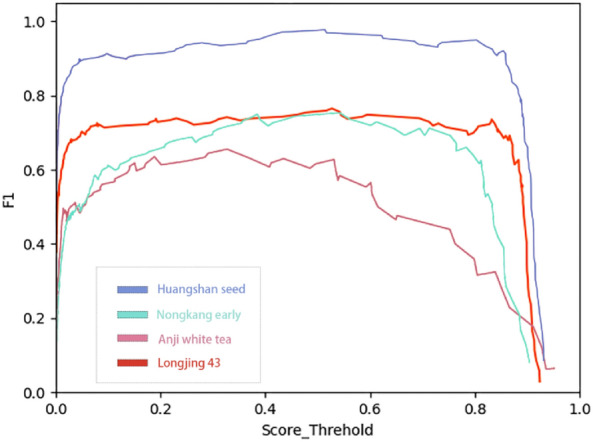
F1 score indicator of YOLOX classification model.

F1 score of the YOLOX classification model is presented in Fig. 8.

**Figure 9:**
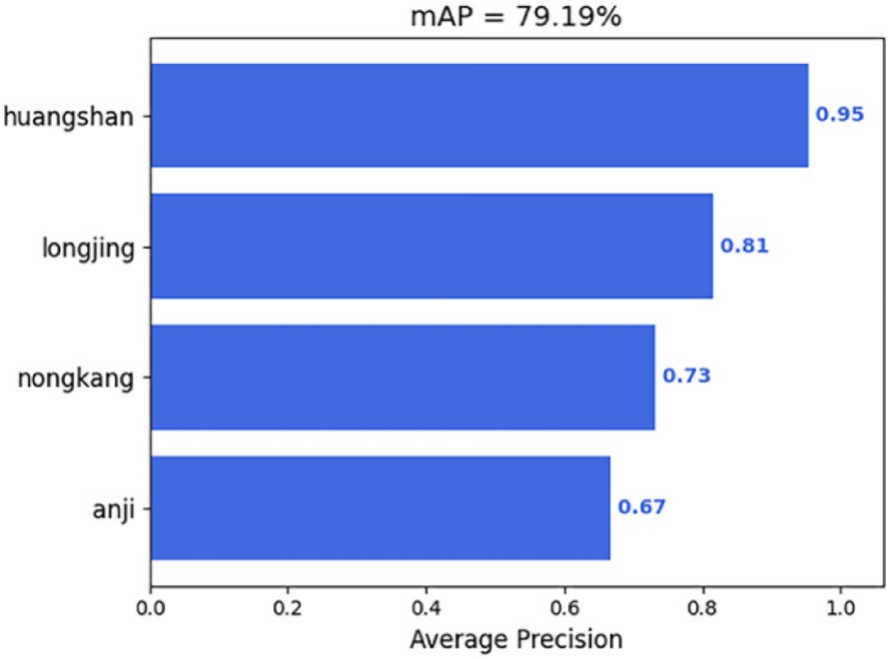
mAP metrics for YOLOX classification models.

mAP of the YOLOX classification model is illustrated in Fig. 9.

Table 4 presents a comparison of the accuracy indicators of Anji white tea, Huangshan species, Longjing 43, and Nongkang early by the YOLOX classification model.

**Table 4:** Summary of accuracy indicators of YOLOX classification model.

Evaluation metrics	Precision (%)	Recall (%)	F1 score	AP (%)	mAP
Anji white tea	76.19	56.67	0.63	67	79.19%
Huangshan seed	90.54	93.06	0.92	95	
Longjing 43	80	77.61	0.76	81	
Nongkang early	78	66.67	0.72	73	

From Table 4, it can be seen that the YOLOX classification model has the highest accuracy in identifying the Huangshan variety, followed by Longjing 43, NongKang Early and Anji White Tea. The accuracy of the YOLOX classification model in identifying the Anji White Tea, Longjing 43, and NongKang Early is closer to the overall accuracy of the YOLOX classification model, and it identifies the NongKang Early as Longjing, which is probably due to the more similarity in the color characteristics of the two varieties of tea shoots, and the highest accuracy in the identification of the Huangshan variety is due to the more noticeable difference in the background color of the shoots and old leaves. The color difference between the background color of the shoots and the old leaves of the Huangshan variety is more prominent, followed by a more significant difference in the color characteristics of the shoots of the Huangshan variety and the other three varieties.

## Discussion

This experiment builds a tea bud recognition model based on seven mainstream algorithms. After detailed comparison experiments, we found that the YOLOX algorithm performed the best. Subsequently, we combined the shoot recognition model with the TBD dataset and successfully created a classification model covering four tea shoot types. The recognition effect of the model is entirely satisfactory, which provides substantial theoretical support for mechanically intelligent tea picking and a solid theoretical foundation for future mechanically intelligent tea picking in practical applications.

Considering that the quality of tea is usually closely related to the time and way of picking, especially for high-grade tea, the quality of the shoots directly affects the quality of tea. Through precise sorting techniques, we can better control the quality of tea leaves and ensure that only high-quality shoots are picked accurately. This helps improve the market competitiveness and added value of tea and positively impacts the entire tea industry.

It is worth noting that in academic research, researchers usually focus more on identifying tea buds and often overlook the importance of their classification. However, studying tea bud classification models is significant to the tea industry and consumers. It improves the accuracy of tea quality identification, promotes scientific research and innovation, and pushes the tea industry to develop in the direction of intelligence. In the next phase of experiments, we will continue and extend the study, aiming to improve the robustness of the model to meet the needs of the tea industry.

## Data Availability

The datasets generated during and/or analyzed during the current study are available from the corresponding author on reasonable request. E-mail: 1604598616@qq.com.
