# Author Correction: YOLOX target detection model can identify and classify several types of tea buds with similar characteristics

**DOI:** 10.1038/s41598-024-56132-z

**Published:** 2024-03-06

**Authors:** Mengdao Yang, Weihao Yuan, Gaojian Xu

**Affiliations:** 1 Anhui Agricultural University, Hefei, 230036 Anhui China; 2 School of Information and Artificial Intelligence, Anhui Agricultural University, Hefei, 230036 China

Correction to: *Scientific Reports* 10.1038/s41598-024-53498-y, published online 03 February 2024

The original version of this article contained an error in the Funding section.

“2020 Anhui University Natural Science Research Key Project/Item Number: KJ2020A0106; 2020 Anhui Province Quality Engineering Project/Item Number: 2020kfkc185.”

now reads:

“A cloud model-based method for determining the grade of wheat blast disease, Item No.: KJ2021721”

The original Article has been corrected.

